# Using media to enhance paediatric patient recruitment for research in primary care

**DOI:** 10.1017/S1463423625100315

**Published:** 2025-07-18

**Authors:** Ilse N. Ganzevoort, Marjolein Y. Berger, Marc A. Benninga, Arine M. Vlieger, Gea A. Holtman

**Affiliations:** 1 Department of Primary and Long-term Care, University of Groningen, University Medical Center Groningen, Groningen, The Netherlands; 2 Department of Pediatrics, Emma Children’s Hospital, Amsterdam University Medical Center, Amsterdam, The Netherlands; 3 Department of Pediatrics, St Antonius Hospital, Nieuwegein, The Netherlands

**Keywords:** Children, irritable bowel syndrome, functional abdominal pain, patient recruitment, primary health care

## Abstract

Recruitment of participants for research is often difficult in primary care, especially children and adolescents. Poor recruitment often leads to extension or discontinuation of randomized controlled trials involving patients. This study describes the impact of media recruitment compared to recruitment via general practitioners (GPs) on characteristics of 152 children aged 7–17 years with functional abdominal pain (FAP) and irritable bowel syndrome. Demographics, clinical and psychosocial characteristics were compared. No clinically relevant differences were found, except for longer pain symptom duration and more diagnoses of FAP in children recruited via media compared to children recruited by their GP. Our results suggest that recruitment via media is effective to recruit children in primary care without inducing relevant baseline characteristic differences and this might decrease research recruitment load for GPs. Subgroup analyses on recruitment method are recommended because recruitment strategy might induce differences in unknown baseline characteristics between groups.

## Introduction

The recruitment of study participants by general practitioners (GPs) is a general concern in primary care research because of time constraints, lack of staff and training. In addition, GPs worry about the impact on their relationship with the patient for participation in research and withhold from engaging in recruiting patients for research (Ross *et al.* 1999; van der Windt *et al.* 2000; Yallop *et al.* 2006; van der Wouden *et al.* 2007; van den Brink *et al.* 2020). Participating GPs often fail in recruiting patients due to low relative incidence of the disease of the study, forgetfulness or time investment (van der Windt *et al.* 2000; van den Brink *et al.* 2020). However, once patients are identified, the next hurdle is the inclusion of the patients themselves. The process of including children as participants in clinical research is especially challenging because informed consent from both parents is needed (Nelson *et al.* 2013). Other factors influencing participation of children and adolescents are the sociodemographic status of the parents, severity of the disease, the study’s characteristics, expectations, and benefits of the intervention studied (Bencheva *et al.* 2024).

Poor recruitment leads to extended periods of data collection in more than half of the studies (van der Wouden *et al.* 2007), and discontinuation in 11% of randomized controlled trials (RCTs) involving patients (Kasenda *et al.* 2014; Speich *et al.* 2022). One solution to enhance recruitment is by using media, e.g. with flyers, newspapers, and paid advertisements on social media platforms (Akard *et al.* 2015; Tsaltskan *et al.* 2023). This approach offers a large and broad reach including hard-to-reach populations, can accelerate inclusion, and reduce recruitment costs (Ngune *et al.* 2012; Whitaker *et al.* 2017; Tsaltskan *et al.* 2023; Tomiwa *et al.* 2024). However, relying on (social) media may reduce practitioner engagement and exclude individuals without internet access, potentially affecting the reliability and generalisability of study results (Wilson *et al.* 2000; Ngune *et al.* 2012; Frandsen *et al.* 2016; Laws *et al.* 2016; Topolovec-Vranic and Natarajan 2016; Tomiwa *et al.* 2024).

An example of a study with recruitment issues is our RCT investigating the (cost-)effectiveness of hypnotherapy in children with functional abdominal pain (FAP) and irritable bowel syndrome (IBS) in primary care. A review showed that practitioner engagement, simplified processes, and minimal workload could boost recruitment efforts (Ngune *et al.* 2012). However, recruiting children by GPs was problematic despite our efforts to minimize workload and actively engage GPs with study visits, telephone calls and newsletters. Therefore, recruitment was extended by also recruiting children with FAP and IBS via media, including social media. In this study, we aim to compare demographic, clinical and psychosocial characteristics of children with FAP or IBS who are recruited by their GP versus via media.

## Methods

### Study design and setting

**Figure 1.**
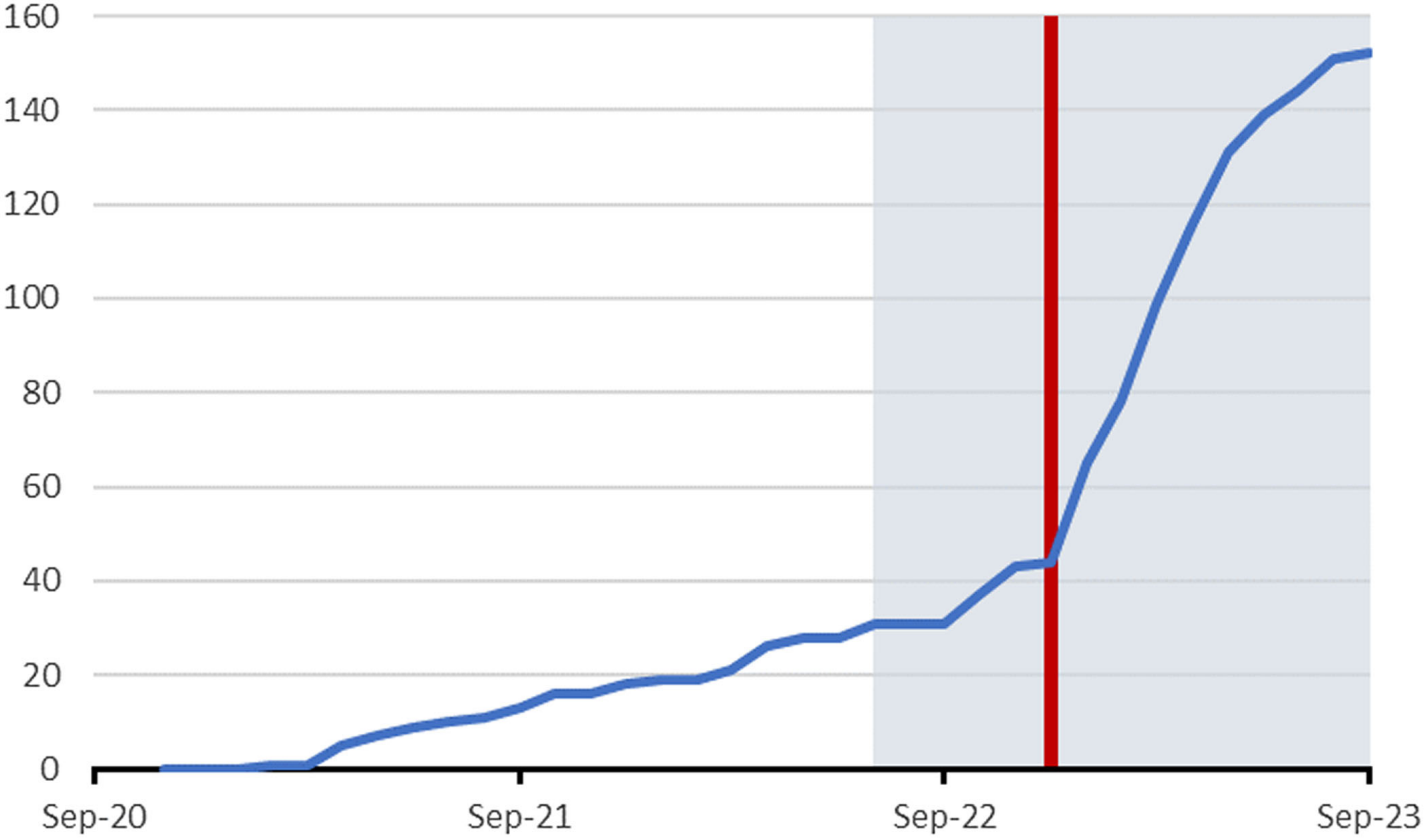
Recruitment trajectory of the trial over time. During the first part of the trial, children were recruited by general practitioners only. During the last part of the trial depicted in the dark area, children were also recruited via media. The red line indicates the start of paid advertisements via social media.

This ancillary study used baseline data from an RCT evaluating the (cost)effectiveness of home-based guided hypnotherapy plus care as usual versus care as usual alone in children with FAP or IBS in primary care. Its study protocol has been described and published in detail (Ganzevoort *et al.* 2023). From November 2020 to September 2023, children were recruited by 94 GPs from 49 practices in the Northern part of the Netherlands. As from July 2022, children were additionally recruited via media because of slow recruitment rates (Figure 1). Media recruitment consisted of spreading information via local schools, different interest groups (e.g., parents and IBS groups), local media (e.g., newspapers and radio), and social media posts (e.g., Facebook, Instagram, Twitter and LinkedIn). From December 2022, this strategy was extended by paid advertisements on Meta (i.e., Facebook and Instagram) throughout the entire country. Detailed information on the advertisements is provided in Supplement 1. The trial protocol and an amendment including extended recruitment strategy were approved by the Medical Ethics Review Committee of the University Medical Center Groningen (METc2020/237). This trial has been registered in ClinicalTrials.gov (ID: NCT05636358).

### Study population

Children were eligible for the trial when they were aged 7–17 years and when their GP suspected FAP or IBS. Exclusion criteria were a concomitant underlying organic gastrointestinal disease, abdominal symptoms treated by a paediatrician, intellectual disability, having a psychotic disorder, a history of hypnotherapy in the past year, and insufficient knowledge of the Dutch language. All children, irrespective of recruitment strategy, were checked on eligibility criteria by their GP. GPs sent the study eligibility criteria forms to the researchers, and the researchers sent information and consent forms to children and parents, and contacted them by phone.

### Baseline characteristics

Baseline characteristics included demographic, clinical, and psychosocial patient characteristics. These were noted during intake meetings and filled out in questionnaires administered before randomization. Demographic characteristics included gender and age. Clinical characteristics included abdominal pain symptom duration, a diagnosis based on Rome IV criteria (Hyams *et al.* 2016), history of treatment by a paediatrician, pain severity, and somatization scores. Psychosocial characteristics included school absenteeism in the past three months, pain beliefs, anxiety and depression scores, and health-related quality of life (HR-QoL). Details of the outcomes and instruments are presented in Table 1.

**Table 1.** Details of outcomes and measurement instruments

Outcome	Instrument	Score calculation and interpretation
Pain severity	Numerical rating scale-11 (NRS-11) (Castarlenas *et al.* 2017)	Severity of pain and/or discomfort in the past week is rated on an 11-point NRS scale from 0 (no pain) to 10 (worst pain). Higher scores indicate higher pain severity.
Somatization	Children’s Somatization Inventory (CSI) (Meesters *et al.* 2003)	Physical symptoms in the previous two weeks are rated on 35 items using a 5-point Likert scale ranging from no problems (score 0) to a lot (score 4). A total score is the sum of all items, with higher scores indicating more somatic complaints (range: 0–140).
Pain beliefs	Pain Beliefs Questionnaire (PBQ) (Stone *et al.* 2016)	A total of 32 items consists of a pain belief statement which range from not true at all (score 0) to very true (score 4). Three subscales are the sum of their items: pain threat (20 items), problem-focused coping efficacy (6 items) and emotion-focused coping efficacy (6 items). A higher score on the pain threat scale indicates stronger belief that their abdominal pain is a threat (range: 0–4). Higher scores on both coping subscales indicate stronger beliefs in their ability to cope with pain (range: 0–4).
Anxiety and depression scores	Revised Anxiety and Depression Scale-short version (RCADS-25) (Muris *et al.* 2002)	This questionnaire comprises 20 items of anxiety and 5 items of depression disorders. Items are rated on a 4-point scale from 0 (never) to 3 (always). A total score for anxiety and depression is the sum of their corresponding items. Higher scores indicate more symptoms of anxiety (range: 0–60) or depression (range: 0–15).
Health-related Quality of Life (HR-QoL)	KIDSCREEN-52 questionnaire (Ravens-Sieberer *et al.* 2005)	Fifty-two items are covered in 10 HR-QoL dimensions: physical well-being, psychological well-being, moods and emotions, self-perception, autonomy, relations with parents and home life, social support and peers, school environment, social acceptance (bullying) and financial resources. Each item is rated on a 5-point Likert scale. Rasch scores are computed for each dimension, and are then converted to T-value norms with a mean of 50 and a standard deviation of ±10. Higher T-values indicate a better HR-QoL and well-being.

### Data analysis

The characteristics of participants in the two recruitment groups were described as proportions for categorical data. Normally distributed continuous data were expressed as mean with standard deviation. For data with non-parametric distribution, median and bootstrapped 95% confidence intervals (CIs) with 1000 repetitions were reported.

## Results

A total of 152 children were included in the RCT, of which 46 by GPs (from 26 practices, range 1–4 per practice) and 106 via media. The most effective strategy turned out to be paid advertisements via Facebook and Instagram, reaching children from across the entire country (Figure 1). Overall, children recruited by GPs and children recruited via media had comparable demographic, clinical and psychosocial characteristics. However, children recruited via media had higher median duration of abdominal pain symptoms (2.5 years, 95% CI 2.1–3.1) compared to children recruited by their GP (1.2 years, 95% CI 0.7–2.6). The proportion of children with FAP according to Rome criteria was higher in children recruited via media (47/106, 44.3%, 95% CI 34.9–53.8), compared to children recruited by their GP (13/46, 28.3%, 95% CI 15.2–41.3). All patient characteristics are presented in Table 2.

**Table 2.** Baseline characteristics of children

	n	GP-recruited children	n	Media recruited children
***Demographic characteristics***				
**Female**, n (%; 95% CI)	46	30 (65.2; 52.2–80.4)	106	67 (63.2; 53.8–71.7)
**Age in years**, median (95% CI)	46	10.0 (9.0–10.9)	106	9.3 (8.9–9.8)
***Clinical characteristics***				
**Symptom duration**, years, median (95% CI)	41	1.2 (0.7–2.6)	101	2.5 (2.1–3.1)
**Rome diagnosis**, n(%; 95% CI)	46		106	
FAP		13 (28.3; 15.2–41.3)		47 (44.3; 34.9–53.8)
IBS		24 (52.2; 39.1–65.2)		49 (46.2; 36.8–55.7)
AM and/or FD		7 (15.2; 4.4–26.1)		9 (8.5; 3.8–14.2)
Not fulfilling Rome criteria*		2 (4.3; 0.0–10.9)		1 (0.9; 0.0–2.8)
**History of visiting a paediatrician**, n (%; 95% CI)	40	8 (20.0; 7.5–32.5)	101	28 (27.7; 18.8–36.6)
**Pain severity**, median (95% CI)	46	5.0 (4.0–6.0)	106	6.0 (5.0–6.0)
**Somatization**, median (95% CI)	45		106	
Total score		16.0 (11.0–17.0)		12.0 (11.0–15.0)
***Psychosocial characteristics***				
**School absenteeism**, n (%; 95% CI)	46	26 (56.5; 41.3–71.7)	106	55 (51.9; 42.5–61.3)
**Pain beliefs**, median (95% CI)	45		106	
Problem-focused coping efficacy		1.5 (1.3–1.7)		1.4 (1.2–1.7)
Emotion-focused coping efficacy		2.0 (1.8–2.5)		2.3 (2.3–2.6)
Pain threat		2.3 (2.0–2.6)		2.2 (2.0–2.3)
**Anxiety and depression scores**, median (95% CI)	45		106	
Anxiety total score		12.0 (10.0–15.0)		11.0 (9.0–13.0)
Depression score		2.0 (2.0–4.0)		3.0 (2.0–3.0)
**HR-QoL**, median (95% CI)	46		106	
Physical well-being		46.5 (43.7–51.1)		48.3 (46.5–49.5)
Psychological well-being		48.9 (48.0–54.9)		48.9 (48.9–52.1)
Moods and Emotions		44.7 (41.2–48.7)		41.8 (38.0–43.9)
Self-perception		49.1 (44.3–54.2)		50.6 (46.5–55.4)
Autonomy		50.9 (48.2–53.9)		51.0 (48.2–53.5)
Parents		52.0 (49.4–55.1)		52.1 (49.4–55.1)
Social or peers		48.4 (46.4–51.9)		53.0 (52.4–55.4)
School		52.1 (46.9–54.5)		52.1 (48.6–54.5)
Social acceptance (bullying)		46.4 (42.2–50.6)		50.6 (44.8–50.6)
Financial resources		61.1 (50.6–62.9)		61.1 (57.8–65.0)

GP = general practitioner; 95% CI = 95% confidence interval; FAP = functional abdominal pain; IBS = irritable bowel syndrome; AM = abdominal migraine; FD = functional dyspepsia; HR-QoL = heath-related quality of life. *Three children did not fulfil Rome criteria (reported 2–3 days of abdominal pain in past 2 months, required ≥4).

## Discussion

### Summary

This study showed that media recruitment and in particular paid social media advertisements, can help attain sample size. Sample characteristics may differ because of different recruitment strategies. This study found differences between groups on abdominal pain symptom duration and diagnosis, with a larger duration and more Rome diagnoses of FAP seen in children who were recruited via media. However, other demographic, clinical and psychosocial characteristics were comparable between groups.

### Strengths and limitations

One strength is that this study used baseline data which were almost complete. This yielded a large sample size and waived the need to impute data which induces uncertainty. Another strength of this study was that all children were checked by their GP on eligibility criteria, thereby enhancing practitioner engagement. The RCT was not designed to compare patient characteristics based on recruitment strategy. Therefore, a limitation of this study is the possibility that relevant patient characteristics such as parental influences and socioeconomic status were not taken into account.

### Comparison with existing literature

A longer abdominal pain symptom duration in children recruited via media compared to GP-recruitment can be explained because they were not actively recruited by GPs and therefore did not recently visit their GP for FAP or IBS. Based on parent report during recruitment calls, some children had consulted a GP in the past, while others had not, though this was not systematically measured with questionnaires. The reasons for not consulting a GP could include dissatisfaction with previous healthcare provided, believing the GP has nothing to offer, or simply learning to cope with the pain over time. Many children are open to alternative treatment and encountering or actively seeking for solutions could make them more willing to respond to this study, especially in those with long-lasting duration of symptoms (Vlieger *et al.* 2008). It is unknown how abdominal pain symptom duration impacts treatment effect and how relevant these differences are. We expect it to be minimal because earlier evidence whether symptom duration affects prognosis is contradictory (Gieteling *et al.* 2011), and other characteristics in this study were similar between groups. However, a secondary multivariable analysis in a hospital-based study evaluating efficacy of hypnotherapy suggested that shorter symptom duration could be a predictor of treatment success at 12 months (Rutten *et al.* 2017). Given the longer symptom duration in the media group, the overall treatment effect could be attenuated. We suggest performing a subgroup analysis by recruitment strategy, as it may not only relate to differences in symptom duration but also reflect unknown baseline characteristics that could affect prognosis or treatment response.

Children recruited via media showed a higher prevalence of FAP compared to those recruited via GPs. We hypothesize that children with changes in defaecation pattern, typical of IBS, are more likely to consult their GP, whereas children with FAP may be less likely to seek medical attention and thus more likely to be reached through media recruitment. However, a hospital-based study evaluating the efficacy of hypnotherapy found no significant effect of diagnosis on treatment success in children with abdominal pain-related disorders of gut-brain interaction (Rutten *et al.* 2017). Therefore, despite the differences in diagnoses between the recruitment groups, we do not expect this to influence the treatment outcomes in our RCT.

Anxiety, depression and HR-QoL scores in both groups were comparable to those found in Dutch and European community samples (Muris *et al.* 2002; Ravens-Sieberer *et al.* 2005). Compared to a paediatric sample, children in this study had lower somatization scores which were more comparable to those in a community sample (Meesters *et al.* 2003). This emphasizes that although children have abdominal pain in primary care, its impact on somatic symptoms and psychosocial characteristics is limited.

This study adds to the literature that Facebook advertisements are effective in recruiting children and adolescents in primary care (Amon *et al.* 2014). Our results are in agreement with other studies performed in primary care evaluating the effect of media recruitment compared to GP-recruitment on patient characteristics in adults. Two studies in adult women in primary care found no relevant differences in sociodemographic and clinical characteristics (Laws *et al.* 2016; van der Worp *et al.* 2020). A systematic review found that online recruitment strategies often lead to differences in age, gender and education in adults, but we did not observe such differences in our study (Brøgger-Mikkelsen *et al.* 2020). Furthermore, our study showed that media recruitment in primary care effectively enhanced study engagement without major changes in the intended study sample characteristics. It enabled broad national reach and, despite financial investment, reduced the recruitment period, making it efficient and potentially cost-effective. Families without internet access may have been missed. While their children might also benefit from hypnotherapy, they would more likely require in-person treatment rather than an online intervention.

### Implications for research

Difficulties in recruiting children for trials in primary care could be overcome by using broader recruitment strategies such as media. Future studies could use such recruitment strategies from the start to gain sufficient sample sizes and statistical power, and to reduce the chance of extended study periods or discontinuation. In addition to reducing the burden on GPs to actively recruit participants for research, media recruitment may facilitate nationwide inclusion and improve cost-efficiency. These aspects could enhance the efficiency and effectiveness of future studies in primary care and help address the paediatric research gap.

## Supporting information

Ganzevoort et al. supplementary material Ganzevoort et al. supplementary material

## Supplementary material

The supplementary material for this article can be found at https://doi.org/10.1017/S1463423625100315
