# Supplementary material for: Using media to enhance paediatric patient recruitment for research in primary care
Source: Prim Health Care Res Dev. 2025 Jul 18;26:e62. doi: 10.1017/S1463423625100315 (PMC12281040; doi:10.1017/S1463423625100315)
Supplement: Ganzevoort et al. supplementary material [file S1463423625100315sup001.docx]

**SUPPLEMENT 1. ADVERTISEMENTS**

**Content of advertisements**

The advertisements provided information about a study investigating a new treatment for abdominal pain. They included a brief description of the study's purpose and a relevant image or photo, along with a link to the study website. The primary aim of the advertisement was to inform the public about the ongoing research. Interested individuals could visit the website for more information and sign up for participation. The advertisements alternated between different designs, each featuring varying images or photos. Four screenshots of the advertisements in Dutch are provided as examples (Figure 1).

**Target population**

Two distinct target populations were identified for the media campaign, with automatic budget allocation between them. The first population targeted parents, specifically women aged 25-55 years in the Netherlands. This group had an estimated size of 126,400 to 148,700 individuals and was selected based on Facebook's “Parents” category. The second population was aimed at a broader audience, with the idea of informing not only parents but also grandparents, aunts, uncles, neighbors, and others who might share the information. This group had an estimated size of 9.1 million to 10.7 million individuals, aged 25-65+ years, also within the Netherlands. Facebook and Instagram (Meta) were the primary platforms used to reach both populations.

**Budget**

The total budget allocated for the media campaign was €5,000. The budget was automatically divided between the two target populations. A bid strategy was used, where the cost was calculated based on cost-per-click for each link click. The advertising set budget determined the amount allocated to each set of ads, with Meta optimizing the delivery of the ads to maximize clicks within the budget.

| 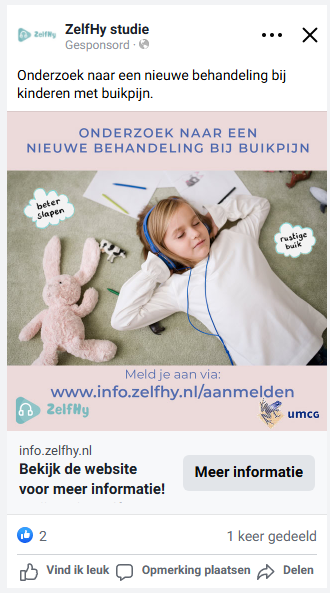 | 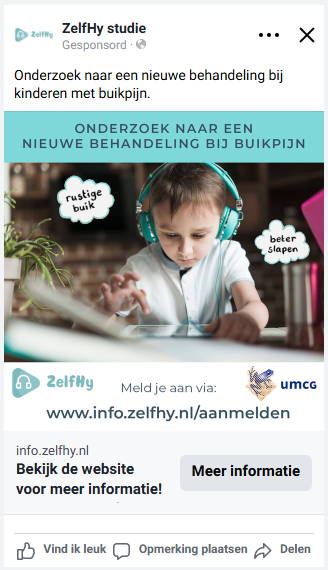 |
| --- | --- |
| 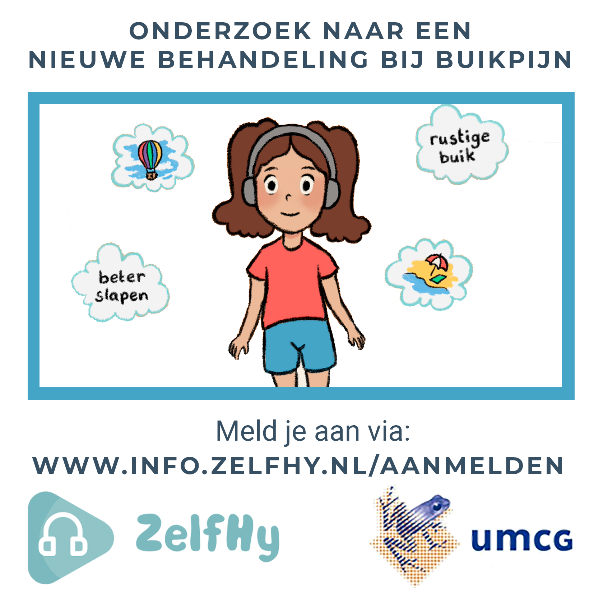 | 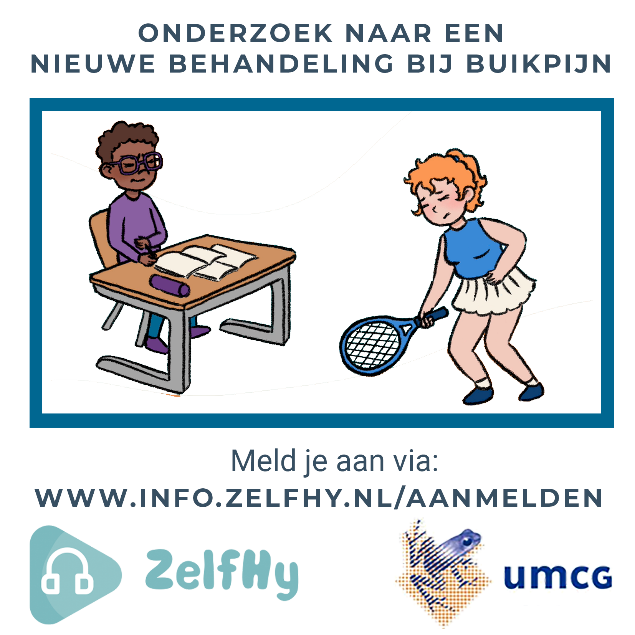 |

**Figure 1. Screenshots of the advertisements**

“Study on a new treatment for abdominal pain” (Dutch: “Onderzoek naar een nieuwe behandeling bij buikpijn”); “Register via: www.info.zelfhy.nl/aanmelden” (Dutch: “Meld je aan via: www.info.zelfhy.nl/aanmelden”); “Visit the website for more information!” (Dutch: “Bekijk de website voor meer informatie!”)
